# Preschool Outcome After Therapeutic Hypothermia in Neonatal Hypoxic-Ischemic Encephalopathy: Insights from Early Neurodevelopmental Assessment

**DOI:** 10.3390/jpm16100509

**Published:** 2026-09-29

**Authors:** Domenico M. Romeo, Sara Curtis, Diego Lopriore, Maria Luisa Piscopiello, Maria Mallardi, Francesca Serrao, Giovanni Vento, Eugenio Mercuri, Claudia Brogna

**Affiliations:** 1 Pediatric Neurology Unit, Fondazione Policlinico Universitario A. Gemelli IRCCS, 00168 Rome, Italy; sara.curtis01@icatt.it (S.C.);; 2 Pediatric Neurology Unit, Università Cattolica del Sacro Cuore, 00168 Rome, Italy; 3 Neonatal Intensive Care Unit, Fondazione Policlinico A. Gemelli IRCCS, 00168 Rome, Italy

**Keywords:** preschool outcome, neonatal hypoxic-ischemic encephalopathy, cognitive development

## Abstract

**Background:** Therapeutic hypothermia has significantly improved the prognosis of infants with hypoxic–ischemic encephalopathy (HIE), shifting the focus of follow-up toward the identification of early predictors of preschool neurodevelopmental outcome. This study aimed to evaluate the predictive value of early neurological, developmental and neuroradiological assessments in children with HIE treated with therapeutic hypothermia. **Methods:** We conducted a prospective longitudinal study including 105 children with HIE treated with therapeutic hypothermia between 2015 and 2022 at a tertiary neonatal center. Early neurological examination using the Hammersmith Infant Neurological Examination (HINE), Griffiths Mental Development Scales at 24 months, brain magnetic resonance imaging (MRI), and neonatal clinical variables were evaluated as predictors of preschool neurodevelopmental outcome. Outcome was classified as favourable or adverse according to cognitive performance and the presence of major neurodevelopmental impairment. **Results:** Of the 105 children included in the preschool follow-up, 84 (80.0%) had a favourable neurodevelopmental outcome and 21 (20.0%) an adverse outcome. Moderate-to-severe neonatal MRI abnormalities were observed in 11/84 (13.1%) children with favourable outcome and 12/21 (57.1%) with adverse outcome (*p* < 0.001). Children with adverse outcomes had lower HINE scores at 12 months (*p* < 0.001) and lower Griffiths Global Developmental Quotients at 24 months (*p* < 0.001). ROC analyses showed high discrimination for HINE (AUC 0.920), Griffiths Global Developmental Quotient (AUC 0.917), and Griffiths Hearing and Speech Scale (AUC 0.915). **Conclusions:** Most children with moderate-to-severe HIE treated with therapeutic hypothermia achieved favorable preschool outcomes. Neonatal MRI, followed by standardized neurological and developmental assessments during infancy and toddlerhood, provides complementary information that may progressively refine neurodevelopmental prognostication.

## 1. What This Paper Adds

(1) Four out of five children treated with therapeutic hypothermia achieved a favourable preschool outcome.
(2) HINE and Griffiths assessments identify children at risk for adverse neurodevelopmental outcomes.
(3) Griffiths Hearing and Speech scores may identify early developmental vulnerabilities warranting further longitudinal assessment.

## 2. Introduction

Perinatal asphyxia remains one of the leading causes of neonatal mortality and long-term neurodevelopmental disability worldwide, with hypoxic-ischemic encephalopathy (HIE) occurring in approximately 1–3 per 1000 live births in high-income countries and even more frequently in low-resource settings [1,2]. Over the last two decades, therapeutic hypothermia (TH) has become the standard treatment for infants with moderate-to-severe HIE and has significantly reduced mortality and severe neurological disability by limiting secondary brain injury mechanisms [3,4]. Consequently, increasing attention has shifted from major disabilities to more subtle neurodevelopmental impairments that may emerge during early childhood despite apparently favourable neonatal recovery.

Several studies have demonstrated that children treated with therapeutic hypothermia may present cognitive, language, behavioural and motor difficulties even in the absence of severe motor impairment [5,6,7,8]. This evolving concept suggests that neurodevelopment after HIE should no longer be considered a dichotomous outcome (normal versus impaired), but rather a continuum in which subtle deficits may become evident only during preschool and school age. Early identification of children at increased risk is therefore essential to guide follow-up programmes and timely intervention.

Neonatal biomarkers, including the severity of encephalopathy, biochemical markers of intrapartum hypoxia, cerebral function monitoring (CFM), and magnetic resonance imaging (MRI), provide important prognostic information during the acute phase [9,10,11]. However, neurodevelopment is a dynamic process, and prognostic information may be progressively refined through standardized neurological and developmental assessments performed during infancy and early childhood. A longitudinal approach integrating information obtained at different developmental stages may therefore provide a more clinically meaningful characterization of individual neurodevelopmental trajectories.

Neurological and developmental assessments performed during infancy may offer additional prognostic information. The Hammersmith Infant Neurological Examination (HINE) is a simple, standardized, and widely validated neurological assessment tool that has demonstrated excellent predictive value for motor outcomes and cerebral palsy in both preterm and term-born high-risk infants [12,13,14,15]. Similarly, the Griffiths Mental Development Scales provide a comprehensive evaluation of early developmental functioning and allow the identification of delays across multiple developmental domains [16]. Whether these early functional assessments provide additional prognostic information beyond neonatal biomarkers remains an important clinical question.

In a previous prospective study conducted at our institution [15], we demonstrated that HINE scores obtained at 12 months were strongly associated with motor and developmental outcomes at 24 months in infants with HIE treated with TH. In that cohort, sub optimal HINE scores were consistently associated with motor impairment, whereas infants with scores above this threshold generally achieved independent ambulation and normal developmental quotients at two years of age. Furthermore, lower HINE scores were associated with more severe patterns of neonatal brain injury on MRI. A subgroup of children with normal global developmental quotients at 24 months showed selective impairments in the hearing and speech domain, suggesting that early developmental assessments may reveal vulnerabilities not captured by global developmental measures alone [15].

However, whether these early neurological and developmental assessments are also predictive of later cognitive and global neurodevelopmental outcomes during preschool age remains unclear. As increasingly favourable outcomes are observed following therapeutic hypothermia, the ability to identify the minority of children who will subsequently develop significant neurodevelopmental impairment becomes particularly important for optimizing follow-up strategies and targeting early intervention programs. The present study extends our previous follow-up cohort by evaluating neurodevelopmental outcomes up to preschool age in children with moderate-to-severe HIE treated with therapeutic hypothermia.

The aim of this study was to examine how neonatal, neurological, developmental and neuroradiological assessments obtained at successive stages of follow-up contribute to the longitudinal prognostication of preschool neurodevelopmental outcome in children with HIE treated with therapeutic hypothermia.

## 3. Materials and Methods

### 3.1. Study Design and Participants

This prospective longitudinal cohort study was conducted at the Pediatric Neurology Unit of Fondazione Policlinico Universitario A. Gemelli IRCCS, Rome, Italy. The study included all consecutive term and near-term infants with hypoxic-ischemic encephalopathy (HIE) treated with therapeutic hypothermia (TH) between January 2015 and May 2022 and subsequently enrolled in a structured multidisciplinary follow-up program.

Infants with a gestational age of ≥36 weeks who developed signs of encephalopathy within the first 6 h of life were considered eligible. Inclusion criteria were based on evidence of intrapartum hypoxia-ischemia and included either: (i) umbilical cord or neonatal blood pH ≤ 7.0 or base deficit ≥ 16 mmol/L within the first hour of life, or (ii) an acute perinatal event associated with an Apgar score ≤ 5 at 10 min or the need for ongoing respiratory support at 10 min after birth [3,4].

All infants underwent a standardized neurological examination within 1 h of life and were classified according to modified Sarnat criteria as having moderate or severe encephalopathy [1,2,3,4]. Infants fulfilling eligibility criteria received whole-body therapeutic hypothermia for 72 h according to international recommendations [3,4].

Exclusion criteria included major congenital malformations, cerebral malformations, metabolic encephalopathies, severe growth restriction (birth weight ≤ 1800 g), inability to initiate therapeutic hypothermia within the recommended therapeutic window, neonatal abstinence syndrome, parental refusal of participation, or moribund infants for whom no further intensive treatment was planned.

All infants underwent neonatal brain MRI according to the local therapeutic hypothermia protocol. Brain MRI was performed between 7 and 14 days of life and was clinically reported by neuroradiologists at our institution. The neonatal MRI protocol included multiplanar 3D TFE, TSE and SE sequences, susceptibility-weighted imaging, diffusion-weighted imaging, and single-voxel proton MR spectroscopy (PRESS, TE = 144 ms). For the purposes of the present study, MRI findings were categorized according to the pattern and severity of hypoxic–ischemic brain injury using the standardized framework proposed by the Newborn Brain Health Working Group of the Canadian Neonatal Network [17]. MRI findings were classified as normal, isolated white matter injury, minimal basal ganglia and cortical involvement, moderate white matter, cortical and basal ganglia involvement, or severe white matter injury with diffuse basal ganglia involvement. For the dichotomous analysis reported in Table 1, the latter two categories were combined and defined as moderate-to-severe MRI abnormalities.

Cerebral function monitoring (CFM) was performed during the neonatal period. Background activity was classified according to the Hellström-Westas classification as normal, moderately abnormal, or severely abnormal [18]. For statistical analyses, CFM findings were dichotomized as normal versus abnormal, with moderately and severely abnormal background patterns included in the abnormal category.

The study was approved by the Ethics Committee of Fondazione Policlinico Universitario A. Gemelli IRCCS (ID 3544). Written informed consent for participation in the longitudinal follow-up study was obtained from the parents or legal guardians of all participants.

### 3.2. Early Neurological and Developmental Assessment

As part of the institutional follow-up protocol, children underwent serial neurological and developmental assessments during infancy. Neurological examination was performed at 12 months of age using the Hammersmith Infant Neurological Examination (HINE), a standardized assessment evaluating cranial nerve function, posture, movements, tone, reflexes, and behaviour [12,13]. The global HINE score ranges from 0 to 78, with scores ≥ 73 considered optimal at 9–12 months and lower scores considered suboptimal [12,13]. Furthermore, auditory function was assessed using auditory brainstem response (ABR) testing. All children included in the study underwent ABR assessment, with results within the normal range in the entire cohort.

Developmental functioning was assessed at 24 months using the Griffiths Mental Development Scales [16]. The Griffiths assessment provides a Global Developmental Quotient (DQ) and domain-specific subscale quotients, including locomotor, personal-social, hearing and speech, eye-hand coordination, and performance domains. Developmental quotients ≥85 were considered normal, scores between 70 and 84 were classified as borderline, and scores < 70 as abnormal.

Particular attention was paid to the Hearing and Speech subscale (Scale C), given previous observations suggesting selective language vulnerability in children with HIE treated with therapeutic hypothermia [15].

### 3.3. Cognitive Assessment at Preschool Age

Preschool cognitive assessments were performed by a qualified psychologist (MM), who was not aware of the results of the previous HINE and Griffiths assessments at the time of cognitive testing. Cognitive functioning was assessed using standardized instruments selected according to the child’s age, developmental profile, language abilities, and suitability for verbally mediated testing. The WPPSI short form was administered to most children, whereas the complete WPPSI-III was used when a more comprehensive cognitive assessment was clinically indicated [19]. The Leiter International Performance Scale-Revised (Leiter-R), a non-verbal measure of cognitive functioning, was administered when language limitations or reduced suitability for verbally mediated testing made a non-verbal assessment more appropriate [20].

Although these instruments differ in their structure and mode of administration, each provides an age-standardized measure of general cognitive functioning. For the purposes of the present study, standardized scores were used to classify cognitive performance according to the same predefined threshold (IQ ≥ 85 vs. <85); absolute scores obtained with the different instruments were not directly compared.

### 3.4. Neurodevelopmental Outcome Classification

The primary outcome was preschool neurodevelopmental status, classified as favourable or adverse. A favourable outcome was defined as cognitive performance within or above the normal range (IQ ≥ 85) in the absence of major neurodevelopmental impairment. An adverse outcome was defined as either (1) neurodevelopmental impairment of sufficient severity to preclude reliable completion of standardized cognitive assessment or (2) cognitive performance below the normal range (IQ < 85) in children who completed standardized testing. Cerebral palsy was classified according to the criteria proposed by Himmelmann and colleagues [21].

### 3.5. Statistical Analysis

Statistical analyses were designed to evaluate the predictive value of early neurological, developmental, neuroradiological and neonatal variables for preschool neurodevelopmental outcome. Descriptive statistics were used to summarize demographic, clinical, neuroradiological, developmental, and cognitive characteristics of the cohort. Continuous variables were reported as mean ± standard deviation or median and interquartile range (IQR), according to data distribution. Categorical variables were expressed as frequencies and percentages. Children were classified into favourable and adverse neurodevelopmental outcome groups. Comparisons between groups were performed using the Mann–Whitney U test for continuous variables and the χ^2^ test or Fisher’s exact test for categorical variables, as appropriate.

Receiver operating characteristic (ROC) curve analyses were performed to evaluate the ability of HINE scores at 12 months and Griffiths Global Developmental Quotient (DQ) and Hearing and Speech Scale (Scale C) scores at 24 months to discriminate between favourable and adverse preschool neurodevelopmental outcomes. Discriminative ability was quantified using the area under the ROC curve (AUC) with 95% confidence intervals calculated using the DeLong method. ROC analyses were restricted to children with available numerical scores for the corresponding assessment. Statistical significance was defined as a two-sided *p* value < 0.05.

## 4. Results

Between January 2015 and May 2022, 124 surviving children with moderate-to-severe HIE treated with TH were enrolled in the institutional prospective follow-up program and reached the minimum age required for preschool neurodevelopmental assessment, and were eligible for the present follow-up study. A total of 19 children were lost to follow-up and preschool outcome data were available for 105 children (85%), who constituted the study population.

### 4.1. Cognitive Outcome

A total of 105 children with HIE treated with therapeutic hypothermia were included in the study. Preschool cognitive assessment was performed at a median age of 3 years and 5 months (range 2 years and 6 months to 6 years). Among the 87 children who completed standardized cognitive testing, 55 were assessed using the WPPSI short form, 22 using the WPPSI-III, and 10 using the Leiter-R.

At preschool follow-up, 84 children (80.0%) had a favourable neurodevelopmental outcome, whereas 21 (20.0%) were classified as having an adverse outcome according to the predefined criteria. Of these, 18 were unable to complete a reliable standardized cognitive assessment because of significant neurodevelopmental difficulties: eleven children had cerebral palsy, two had autism spectrum disorder, and two had mixed specific developmental disorders (ICD-10 F83). One child with attention-deficit/hyperactivity disorder had severe attentional difficulties and motor hyperactivity that prevented reliable test administration; two additional children had undergone genetic testing because of dysmorphic features, which identified rare genetic variants not associated with a specific genetic syndrome considered explanatory of their neurodevelopmental phenotype. The remaining three children completed standardized cognitive testing but obtained scores below the normal range (IQ < 85). (Table 1). Numerical Griffiths scores were available for 103 of 105 children; two children with severe neurodevelopmental impairment were non-evaluable. Both subsequently belonged to the adverse-outcome group. Excluding these two children from the categorical analysis, Global Developmental Quotient < 85 was observed in 0/84 children with favourable outcome and 14/19 with adverse outcome (*p* < 0.001). Hearing and Speech Scale scores < 85 were observed in 26/84 and 16/19 children, respectively (*p* < 0.001). No child had blindness or a major visual impairment that precluded standardized neurodevelopmental or cognitive assessment.

Cognitive performance was predominantly within the normal range (Table 2). Seventeen children (19.5%) achieved scores above the expected normative range, 67 (77%) scored within the average range, and two (2.3%) obtained borderline scores. Only one child (1.1%) met criteria for mild intellectual disability. This child also presented with a Chiari type I malformation.

### 4.2. Neonatal Predictors

Neonatal brain MRI was available for all children included in the outcome analysis and was classified according to the predominant pattern of injury. The distribution of MRI findings according to neurodevelopmental outcome is reported in Table 3. Normal MRI findings and isolated white matter abnormalities were predominantly observed among children with favourable outcomes. Conversely, severe patterns of injury involving diffuse basal ganglia and white matter abnormalities were strongly associated with adverse neurodevelopmental outcome. Overall, increasing MRI severity was associated with a higher risk of adverse neurodevelopmental outcome. However, of the 21 children with adverse outcome, 5 reported normal MRI and 3 isolated white matter injury; these 8 children presented with autism, neurodevelopmental disorders or low cognitive scores; one only presented with CP.

Neonatal biochemical markers demonstrated only limited discriminatory ability when the entire spectrum of adverse neurodevelopmental outcomes was considered. Nevertheless, children who subsequently developed cerebral palsy tended to show lower cord blood pH values compared with the remainder of the cohort, suggesting that markers of perinatal acidosis may be more closely associated with severe motor outcomes than with later cognitive functioning (Table 1).

### 4.3. Early Predictors of Neurodevelopmental Outcome

Children with adverse neurodevelopmental outcomes showed significantly lower Hammersmith Infant Neurological Examination (HINE) scores at 12 months compared with those who achieved favorable outcomes (Table 1). The median HINE score was 76 in the favourable outcome group and 66 in the adverse outcome group (*p* < 0.001). Children with favourable outcomes had a median Griffiths Developmental Quotient of 114, compared with 67 among children with adverse outcomes (*p* < 0.001). The Hearing and Speech subscale (Scale C) of the Griffiths assessment also showed a marked relationship with preschool outcome. Mean Scale C scores were substantially higher in children with favorable outcomes than in those with adverse outcomes (93 vs. 54, respectively).

Children who were subsequently assessed using the non-verbal Leiter-R had lower Griffiths Hearing and Speech Scale scores at 24 months than children assessed using verbally mediated cognitive measures. However, because the Leiter-R was preferentially selected for children in whom a non-verbal assessment was considered more appropriate, this association should be interpreted cautiously and may partly reflect the test-selection process.

Suboptimal HINE scores (<73) were present in 86% of children with adverse outcomes, while moderate to severe MRI abnormalities were observed in only 43%. Similarly, pathological Griffiths assessments were present in more than three quarters of children with adverse outcomes. Conversely, moderate to severe MRI abnormalities were also observed in a proportion of children who ultimately achieved favorable outcomes.

Receiver operating characteristic (ROC) analyses (Figure 1) showed high discrimination between favourable and adverse preschool neurodevelopmental outcomes for HINE at 12 months (AUC 0.920, 95% CI 0.832–1.000; *n* = 105), Griffiths Global Developmental Quotient at 24 months (AUC 0.917, 95% CI 0.823–1.000; *n* = 103), and Griffiths Hearing and Speech Scale (Scale C) (AUC 0.915, 95% CI 0.849–0.982; *n* = 103). The two children without available numerical Griffiths scores were excluded from the corresponding ROC analyses.

Children with adverse outcomes had markedly lower HINE scores at 12 months and lower Griffiths developmental quotients at 24 months compared with those achieving favourable outcomes.

## 5. Discussion

In this prospective longitudinal cohort of children with moderate-to-severe hypoxic-ischemic encephalopathy treated with therapeutic hypothermia, the majority of patients achieved favourable preschool neurodevelopmental outcomes. Among the 105 children with available outcome data, 80% demonstrated age-appropriate cognitive functioning, while only a minority developed major neurodevelopmental impairment or cognitive performance below the normal range. These findings are consistent with the substantial improvements in survival and neurodevelopmental prognosis reported since the introduction of therapeutic hypothermia as standard care for neonatal HIE [3,4]. Our findings support a longitudinal interpretation of prognostication after HIE. Neonatal clinical variables and MRI provide prognostic information during the acute phase, whereas subsequent standardized neurological and developmental assessments allow this information to be progressively refined as the child’s developmental trajectory becomes apparent. HINE at 12 months and Griffiths assessment at 24 months showed strong associations with preschool outcome, highlighting the value of serial assessment rather than suggesting superiority of later over earlier prognostic measures.

Our results are consistent with previous studies demonstrating that the HINE is a reliable, inexpensive and easily applicable neurological examination capable of identifying infants at increased risk for adverse neurodevelopmental outcomes [12,13,14,15]. Because it assesses functional neurological performance rather than the severity of the initial hypoxic–ischemic insult, the HINE may better reflect the child’s evolving neurodevelopmental trajectory and therefore provide clinically meaningful prognostic information during infancy. Furthermore, the prognostic value of HINE extends beyond early motor outcomes and remains highly informative for predicting neurodevelopmental status several years later as also reported in previous studies in preterm and term born infants [22,23]. This supports the concept that neurological examination during infancy reflects the emerging functional organization of the developing brain.

Similarly, the Griffiths Mental Development Scales showed a strong association with cognitive outcome, particularly the Global Developmental Quotient and the Hearing and Speech Scale. Unlike neonatal biomarkers, standardized developmental assessments evaluate the functional expression of early brain injury across multiple developmental domains. Developmental testing at 24 months therefore provides additional information at a later stage of the child’s developmental trajectory, complementing prognostic information obtained during the neonatal period and infancy. Our findings further support the incorporation of standardized developmental assessment into routine follow-up programmes for infants with HIE treated with therapeutic hypothermia.

Among neonatal investigations, moderate to severe MRI abnormalities remained significantly associated with adverse preschool outcome. This finding is consistent with the well-established role of MRI as the most reliable neuroradiological predictor after HIE [9,10,11]. However, our results also suggest that MRI and early functional assessments should not be regarded as alternative prognostic tools but rather as complementary components of a longitudinal prognostic approach. While MRI characterizes the extent and distribution of brain injury shortly after birth, HINE and Griffiths provide information on the functional consequences of that injury during subsequent development.

Cord blood pH, base excess and CFM provide clinically relevant information on the severity of the initial hypoxic–ischemic event. Their role should therefore be interpreted within the timing at which they are obtained. As development progresses, neurological and developmental assessments provide additional information on the functional consequences of the initial injury and allow prognostic estimates to be progressively refined.

An additional and particularly interesting finding concerns language development. In our previous cohort, several children with normal global developmental quotients already demonstrated selective weaknesses in the Hearing and Speech domain of the Griffiths Scales [15]. The present findings further support the potential relevance of this observation. Griffiths Scale C scores were strongly associated with later neurodevelopmental outcome and showed predictive performance comparable to that of the global developmental quotient.

The association between lower Griffiths Hearing and Speech scores at 24 months and subsequent use of a non-verbal cognitive assessment is of potential interest but should be considered hypothesis-generating. The Leiter-R was preferentially administered when a non-verbal assessment was considered more appropriate, introducing an inherent selection effect. Moreover, a uniform language-specific assessment was not performed at preschool age. Therefore, these findings cannot establish persistence of a specific language impairment from toddlerhood to preschool age, but may support the value of further prospective investigation using standardized longitudinal language assessments. Similar observations have been reported in previous studies describing language and communication difficulties despite otherwise favourable developmental outcomes after therapeutic hypothermia [5,6,7,8]. Further studies specifically focusing on language trajectories and communication skills are needed.

From a clinical perspective, our findings have important implications for follow-up programs. Current follow-up protocols often focus primarily on the identification of major neurological disabilities, particularly cerebral palsy. However, our results suggest that structured neurological and developmental assessments during infancy may also identify children at risk for later cognitive and language difficulties. Children presenting with suboptimal HINE scores or reduced Griffiths developmental quotients may benefit from intensified surveillance and early intervention strategies, even in the absence of overt motor impairment. Taken together, our findings support a longitudinal model of prognostication after HIE, in which neonatal MRI provides information on the severity of brain injury, while serial neurological and developmental assessments progressively refine the prediction of preschool outcome.

The strengths of this study include the relatively large cohort of children treated with therapeutic hypothermia, the standardized longitudinal follow-up programme and the integration of neurological, developmental and neuroradiological assessments performed at predefined time points. Nevertheless, some limitations should be acknowledged. This was a single-centre study, and cognitive outcome was assessed using different standardized instruments according to age and clinical characteristics. Although these instruments differ in structure, all are validated measures of global cognitive functioning and were used according to the child’s language and developmental profile. Furthermore, preschool follow-up may not identify all subtle cognitive and behavioural difficulties that can emerge later during school age; the study was not designed to comprehensively characterize behavioural or emotional outcomes, and standardized behavioural and affective measures were not systematically included. Therefore, these domains should be specifically investigated in future longitudinal studies. A further limitation is the loss to preschool follow-up of 19 of 124 eligible children (15%). As complete baseline data for these children were not available in the study database used for the present analysis, differences between participants retained in the study and those lost to follow-up could not be assessed. Therefore, potential attrition bias cannot be excluded, particularly with regard to the observed proportion of favourable preschool outcomes.

In conclusion, most children with moderate-to-severe HIE treated with therapeutic hypothermia achieved favourable neurodevelopmental outcomes at preschool age, although adverse outcomes remained present in approximately one fifth of the cohort. Our findings support a longitudinal model of prognostication in which neonatal clinical and neuroradiological information is progressively complemented by standardized neurological and developmental assessments during infancy and toddlerhood. Serial assessment with MRI, HINE and Griffiths may help refine individual risk stratification and identify children who require closer developmental surveillance and early intervention.

## Figures and Tables

**Figure 1:**
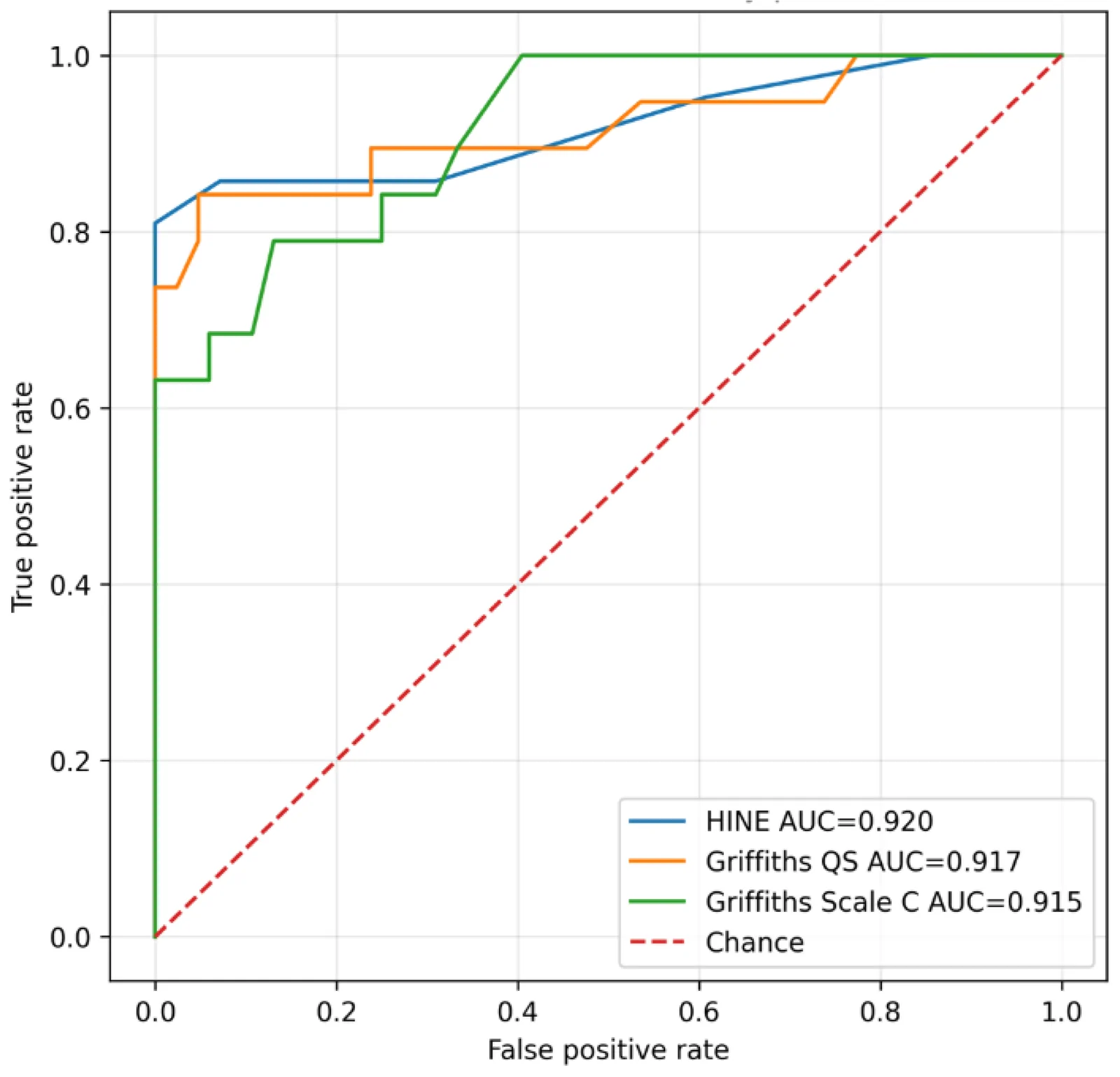
Receiver operating characteristic (ROC) curves for HINE score at 12 months, Griffiths Global Developmental Quotient (DQ), and Griffiths Hearing and Speech Scale (Scale C) at 24 months for discrimination between favourable and adverse preschool neurodevelopmental outcomes. HINE: AUC 0.920 (95% CI 0.832–1.000; *n* = 105); Griffiths Global DQ: AUC 0.917 (95% CI 0.823–1.000; *n* = 103); Griffiths Scale C: AUC 0.915 (95% CI 0.849–0.982; *n* = 103). AUC, area under the curve.

**Table 1:** Early neurological, developmental, neuroradiological and neonatal characteristics according to neurodevelopmental outcome.

Variable	Favorable Outcome (*n* = 84)	Adverse Outcome (*n* = 21)	*p* Value
Male sex, *n* (%)	57/84 (67.9%)	15/21 (71.4%)	1.000
Cord blood pH, median [IQR]	7.03 [6.9–7.2]	6.9 [6.9–7.1]	0.567
Base excess, median [IQR]	−16.0 [−19.5 to −12.8]	−15.8 [−18.7 to −12.0]	0.176
Sarnat stage III, *n* (%)	13/84 (15.5%)	3/21 (14.3%)	1.000
Abnormal CFM, *n* (%)	69/84 (82.1%)	19/21 (90.5%)	0.514
Moderate to Severe MRI abnormalities *, *n* (%)	11/84 (13.1%)	12/21 (57.1%)	<0.001
HINE at 12 months, median [IQR]	76.0 [75.0–77.0]	66.0 [58.0–68.0]	<0.001
HINE < 73, *n* (%)	6/84 (7.1%)	18/21 (85.7%)	<0.001
Griffiths Global DQ, median [IQR]	113.8 [107.1–128.0]	67.0 [61.5–84.9]	<0.001
Griffiths Global DQ < 85, *n*/N (%)	0/84 (0.0%)	14/19 (73.7%)	<0.001
Griffiths Scale C, median [IQR]	93.0 [78.6–108.3]	54.2 [50.0–69.0]	<0.001
Griffiths Scale C < 85, *n*/N (%)	26/84 (31.0%)	16/19 (84.2%)	<0.001

* Moderate-to-severe MRI abnormalities included moderate white matter, cortical and basal ganglia involvement and severe white matter injury with diffuse basal ganglia involvement.

**Table 2:** Cognitive outcomes.

Test	*n*	Mean IQ (SD)	Above Average	Average	Borderline	Intellectual Disability
WPPSI short	55	109 (15.5)	14	40	1	0
WPPSI-III	22	101 (9.6)	2	19	1	0
Leiter-R	10	95 (15.5)	0	9	0	1
Total	87	—	17	67	2	1

**Table 3:** MRI pattern according to the outcome.

MRI Pattern	Favorable Outcome	Adverse Outcome	Total
Normal MRI	36 (88%)	5	41
Isolated white matter injury	25 (89%)	3	28
Minimal basal ganglia and cortical involvement	12 (92%)	1	13
Moderate white matter, cortical and basal ganglia involvement	10 (59%)	7	17
Severe white matter and diffuse basal ganglia injury	1 (17%)	5	6
Total	84 (80%)	21	105

## Data Availability

The data presented in this study are available on request from the corresponding author due to ethical restrictions.
